# Controlled Complexity: Optimized Systems to Study the Role of the Gut Microbiome in Host Physiology

**DOI:** 10.3389/fmicb.2021.735562

**Published:** 2021-09-27

**Authors:** Robert W. P. Glowacki, Morgan J. Engelhart, Philip P. Ahern

**Affiliations:** ^1^Department of Cardiovascular and Metabolic Sciences, Lerner Research Institute, Cleveland Clinic, Cleveland, OH, United States; ^2^Cleveland Clinic Lerner College of Medicine, Case Western Reserve University, Cleveland, OH, United States; ^3^Center for Microbiome and Human Health, Cleveland Clinic, Cleveland, OH, United States

**Keywords:** microbiome, model system, synthetic communities, gnotobiotic, wild mice, translation, model microbial communities

## Abstract

The profound impact of the gut microbiome on host health has led to a revolution in biomedical research, motivating researchers from disparate fields to define the specific molecular mechanisms that mediate host-beneficial effects. The advent of genomic technologies allied to the use of model microbiomes in gnotobiotic mouse models has transformed our understanding of intestinal microbial ecology and the impact of the microbiome on the host. However, despite incredible advances, our understanding of the host-microbiome dialogue that shapes host physiology is still in its infancy. Progress has been limited by challenges associated with developing model systems that are both tractable enough to provide key mechanistic insights while also reflecting the enormous complexity of the gut ecosystem. Simplified model microbiomes have facilitated detailed interrogation of transcriptional and metabolic functions of the microbiome but do not recapitulate the interactions seen in complex communities. Conversely, intact complex communities from mice or humans provide a more physiologically relevant community type, but can limit our ability to uncover high-resolution insights into microbiome function. Moreover, complex microbiomes from lab-derived mice or humans often do not readily imprint human-like phenotypes. Therefore, improved model microbiomes that are highly defined and tractable, but that more accurately recapitulate human microbiome-induced phenotypic variation are required to improve understanding of fundamental processes governing host-microbiome mutualism. This improved understanding will enhance the translational relevance of studies that address how the microbiome promotes host health and influences disease states. Microbial exposures in wild mice, both symbiotic and infectious in nature, have recently been established to more readily recapitulate human-like phenotypes. The development of synthetic model communities from such “wild mice” therefore represents an attractive strategy to overcome the limitations of current approaches. Advances in microbial culturing approaches that allow for the generation of large and diverse libraries of isolates, coupled to ever more affordable large-scale genomic sequencing, mean that we are now ideally positioned to develop such systems. Furthermore, the development of sophisticated *in vitro* systems is allowing for detailed insights into host-microbiome interactions to be obtained. Here we discuss the need to leverage such approaches and highlight key challenges that remain to be addressed.

## Introduction

As a species, humans are surrounded by and inhabited by trillions of microorganisms, encompassing bacteria, fungi, archaea, other eukaryotic organisms such as parasites and protists, as well as viruses (Gill et al., 2006; Parfrey et al., 2011; Hallen-Adams and Suhr, 2017; Koskinen et al., 2017; Nkamga et al., 2017; Gregory et al., 2020) that are collectively referred to as the microbiome. Decades of work have established the profound role of the microbiome in shaping host physiology and its capacity to regulate a wide variety of health and disease states. The rapid growth of microbiome research, spurred by technological innovations, has resulted in remarkable discoveries that have altered our conceptualization of the role played by this complex ecosystem in host health. Furthermore, these efforts have uncovered several features that highlight the therapeutic potential of the microbiome. First, dysfunction of the microbiome or host responses to the microbiome have been implicated in the pathogenesis of myriad human diseases, including, undernutrition and its associated maladies (Smith M. I. et al., 2013; Kau et al., 2015; Blanton et al., 2016; Charbonneau et al., 2016; Wagner et al., 2016; Cowardin et al., 2019), metabolic diseases such as obesity (Ley et al., 2005; Turnbaugh et al., 2006, 2008, 2009b; Hildebrandt et al., 2009; Ridaura et al., 2013), cardiovascular disease (Kelly et al., 2016), cancer and its susceptibility to treatment (Arthur et al., 2012; Buc et al., 2013; Sivan et al., 2015; Vétizou et al., 2015), food allergy (Feehley et al., 2019), multiple sclerosis (Berer et al., 2017; Cekanaviciute et al., 2017), and inflammatory bowel disease (IBD) (Manichanh et al., 2006; Frank et al., 2007; Gevers et al., 2014; Britton et al., 2019). Second, there is significant inter-personal variation in microbiome composition and/or function across individuals (Turnbaugh et al., 2009a; Qin et al., 2010; Schloissnig et al., 2013) that can impact host phenotypes, and thus, microbiome composition represents a personalized risk factor for the development of disease (Smith M. I. et al., 2013; Subramanian et al., 2014; Alavi et al., 2020). Third, microbiota repair, where specific microbial taxa or microbial consortia are introduced to communities lacking these microbes, has proven effective in restoration of beneficial microbiome-mediated effects (van Nood et al., 2013; Buffie et al., 2015; Blanton et al., 2016; Caballero et al., 2017; Di Luccia et al., 2020), underscoring the potential of microbiome manipulation for therapy.

This has prompted a flurry of exploration from researchers across a wide-array of disciplines to provide a systematic understanding of the microbiome and its interaction with the host, especially in defining the features that shape microbiome composition and function, as well as uncovering how the microbiome imparts its beneficial or deleterious effects on host physiology. Investigation of these processes has typically followed a trajectory beginning with identifying disruptions to microbiome composition, commonly referred to as dysbiosis, followed by *in vivo* animal studies whereby transplantation of microbiomes from donors exhibiting a phenotype of interest is used to assess how much, if any, of the donor phenotype can be transmitted by the microbiome. These studies are essential to establish a causal role for the microbiome and microbe(s) in question. This process is exemplified by studies of malnutrition (obesity and undernutrition) and IBD. Obesity is associated with an altered gut microbiome composition (Ley et al., 2005; Turnbaugh et al., 2008, 2009b; Hildebrandt et al., 2009), and the gut microbiome from these individuals or obese mice promotes increased adiposity and metabolic dysfunction upon transplantation to germ-free recipient mice relative to healthy donor controls (Turnbaugh et al., 2006; Ridaura et al., 2013). Likewise, individuals suffering from undernutrition have disrupted gut microbiomes, and transplantation of fecal microbiomes from such donors recapitulates features of weight loss/cachexia in recipient mice relative to control donors (Smith M. I. et al., 2013; Kau et al., 2015; Blanton et al., 2016; Wagner et al., 2016). Although there remains some debate about whether or not IBD patients have distinct microbiome compositions, gut microbiomes from IBD patients elicit more severe intestinal inflammation in gnotobiotic IBD models than those from healthy controls (Gevers et al., 2014; Britton et al., 2019; Lloyd-Price et al., 2019). In addition to establishing a causal role for the microbiome in such diseases, model systems have also been leveraged to elucidate how specific microbiome members impact the progression or prevention of diseases. These efforts have yielded detailed insights that would have been likely impossible without a tractable model system. For example, particular strains of *E. coli* containing the pathogenicity island *pks* have been identified as being enriched in patients with colorectal cancer, and gnotobiotic mouse models have been utilized to demonstrate causality for these specific strains in the disease (Arthur et al., 2012; Buc et al., 2013). Enterotoxigenic strains of *B. fragilis* have also been linked to colorectal cancer development (Toprak et al., 2006; Wu et al., 2009), as well as aspects of undernutrition (Wagner et al., 2016). Species of *Clostridium* and the *Bacteroides* have also been implicated in limiting the severity of food allergy (Atarashi et al., 2011; Stefka et al., 2014; Abdel-Gadir et al., 2019). Animal model systems have thus proven essential in determining causal roles for the microbiome in shaping disease susceptibility and facilitating the precise delineation of host-microbiome interactions that mediate these effects on host physiology. As such, they remain an irreplaceable component of the microbiome researcher’s toolkit.

While the advances to date have been captivating, there is still a dearth of information regarding the specific gut microbes that mediate the effects of the microbiome on the host. Moreover, despite the enormous progress that they have facilitated, current models have deficiencies that limit their translational relevance. As targeted and rational microbiome manipulation becomes an increasingly attractive approach for therapy, tractable and physiologically relevant model systems to interrogate host-microbiome interactions are needed. Here we will discuss current challenges and describe a path to addressing this need in microbiome research through the creation of new and improved model systems to interrogate host-microbiome interactions. We will focus our attention primarily on the effects of gut bacteria on host physiology. However, it is increasingly clear that intestinal fungi, viruses, archaea, and other eukaryotic species can profoundly impact host phenotypes, such as promoting intestinal immune system maturation and regulating disease susceptibility, often able to imprint phenotypic responses equivalent to gut bacteria (Kernbauer et al., 2014; Chudnovskiy et al., 2016; Escalante et al., 2016; Lin et al., 2020; Yeung et al., 2020; Dallari et al., 2021). Moreover, these agents do not act in isolation, and their direct or indirect interactions may regulate host health as has been demonstrated in murine models of inflammatory bowel disease (IBD) and parasitic infection (Cadwell et al., 2010; Hayes et al., 2010). It would therefore be remiss to ignore the contributions of these oft-overlooked microbiome members in our conceptualization of the gut ecosystem and its effects on the host, as highlighted by others (Norman et al., 2014; Reynolds et al., 2015; Runge and Rosshart, 2021).

### Mouse Models for the Study of Host-Microbiome Interactions

Model systems have been widely employed by researchers going all the way back to the days of Gregor Mendel’s use of pea plants to study inherited traits (Ellis et al., 2011). In order to define paradigms of host-microbiome mutualism, researchers have utilized a variety of organisms (Reyniers and Sacksteder, 1958; Smith et al., 2007; Ericsson, 2019), ranging from Drosophila (Lee and Brey, 2013), Hydra (Augustin et al., 2012), zebrafish (Kanther and Rawls, 2010; Stagaman et al., 2020) and squid (Nyholm and McFall-Ngai, 2004; McFall-Ngai, 2014) to mice and rats (Reyniers and Sacksteder, 1958; Smith et al., 2007), and pigs (Vlasova et al., 2018; Gehrig et al., 2019). This has enabled fundamental insights into the relationship between the host and the resident microbiome and the identification of features that typify these interactions, akin to Koch’s postulates that describe the paradigm that defines microbial pathogenesis (Neville et al., 2018). Systems like Drosophila, Hydra, squid, and zebrafish offer numerous advantages including the relative ease of husbandry, the ability to study large numbers of offspring, less complex and more readily cultivated microbiomes for study than higher organisms, the availability of whole-organism imaging, etc. Pioneering studies in these systems have uncovered principles that govern host-microbiome interactions, including (but not limited to): (i) a role for gut symbionts in the coordination of tissue developmental programs and the microbial components responsible for these effects (particularly microbial cell wall products such as peptidoglycan and LPS, as well as microbial metabolites like acetate) (Koropatnick et al., 2004; Buchon et al., 2009; Shin et al., 2011; Troll et al., 2018), (ii) host adaptations to the microbiome that limit the inflammatory potential of microbiota-derived factors (Bates et al., 2007; Lhocine et al., 2008; Rader et al., 2012), (iii) host regulation of microbiome composition (Rawls et al., 2006; Ryu et al., 2008; Fraune et al., 2009), (iv) gut symbiont factors driving host adaptation (Rawls et al., 2007; Koehler et al., 2018), and (v) microbiome contributions to growth and nutrient acquisition (Storelli et al., 2011; Semova et al., 2012; Schwarzer et al., 2016). Gnotobiotic pigs have now become more widely utilized, which has allowed the study of these processes in an animal system with physiology more similar to that of humans than provided by commonly used murine models. While pig models present many challenges due to their size, they offer several advantages over more commonly used model systems, including more human-like physiology, susceptibility to many human-relevant infectious agents, greater microbiome complexity, and therefore they offer important insights of more translational relevance (Vlasova et al., 2018; Ericsson, 2019; Gehrig et al., 2019). Although less commonly employed due to the more challenging and expensive nature of their husbandry, gnotobiotic pig models are proving to be a highly valuable component of microbiome research.

Despite the utility of these other model systems, the mouse has reigned supreme in biomedical research, especially for microbiome studies. The emergence of the mouse as a model organism can be traced to the early 1900s with the house mouse, *Mus musculus* being used to study Mendelian genetics (Castle and Little, 1910), followed shortly thereafter by the development of the first inbred *Mus musculus* strain in 1929 by C.C. Little at what is now known as Jackson labs (Phifer-Rixey and Nachman, 2015). Although models in other small animals have been widely used, including rats (Modlinska and Pisula, 2020), hamsters (Miao et al., 2019), and gerbils (Bleich et al., 2010), none are quite as adapted for the breadth of study possible with mice. Mice offer several advantages that include their relatively quick gestation period, their size, which allows for easier housing and manipulation, the plethora of tools for phenotypic assessment of the mouse, the availability of sophisticated tools for genetic modification to interrogate the role played by distinct genes and cell types, and most importantly the availability of approaches to raise mice in germ-free settings. Furthermore, the availability of inbred strains of mice and standardized, albeit imperfect, housing and husbandry that helps to minimize unwanted variation, allows for easier comparison of data from different researchers.

However, mirroring the interpersonal variation in human microbiomes, model organisms display significant variation in the composition of their microbiomes, which in turn contributes to phenotypic variation reported in mouse models, especially in studies associated with immune activation. Several notable examples highlight how microbiome variation can impact phenotypes in murine models: (i) microbiome mediated spontaneous colitis and metabolic dysfunction has been reported in TLR5-/- mice by some, but not by others (Vijay-Kumar et al., 2007, 2010; Letran et al., 2011); (ii) the aggravation of colitis and development of communicable disease in NLRP6-/- mice is critically dependent on the microbiome context in which it is studied (Elinav et al., 2011; Lemire et al., 2017; Mamantopoulos et al., 2017); (iii) IL-10-/- mice develop a spontaneous colitis in some animal facilities, yet they remain largely free of disease in others depending on the presence or absence of select microbes (Kuhn et al., 1993; Nagalingam et al., 2013; Seregin et al., 2017); (iv) DSS-induced colitis models display significantly varied kinetics and severity depending on microbiome composition (Forster et al., 2021); (v) diabetes in the Non-Obese Diabetic (NOD) type 1 diabetes model is regulated by various parameters, including the presence of specific gut microbes or infectious agents (Wilberz et al., 1991; Takei et al., 1992; Pozzilli et al., 1993; Kriegel et al., 2011; Markle et al., 2013; Yurkovetskiy et al., 2013). Although not an exhaustive list, these examples demonstrate that variation in the gut microbiome can lead to disparate phenotypic outcomes in mice. Experimental variation, whether it be technical or biological in origin, has long been seen as a thorn in the side of researchers, contributing in part to issues of reproducibility in science (von Kortzfleisch et al., 2020). To limit the impact of overt pathogens, and the large-scale microbiome variation across different animal facilities, efforts were made to create a more standardized murine system that would limit issues of reproducibility. Thus, the concept of Specific Pathogen Free (SPF) mice was born. SPF mice are free of certain (but not all) pathogenic organisms or microorganisms capable of interfering with experimental outcomes (Lane-Petter, 1962). These mice provide the advantage of controlling the health status of the animal and allowing for better standardization between experiments, labs, and institutions. While the SPF mouse was adopted with the intention of allowing for more reproducible results, it has been shown that microbiome and phenotypic variability also exists between SPF mouse colonies from commercial vendors, as SPF only determines what is excluded, but not what should be present (Smith et al., 2007; Ivanov et al., 2009; Denning et al., 2011; Rosshart et al., 2017).

**Box 1. Common terminology used to describe the colonization status/microbiome communities commonly found in murine model systems.**

**Germ-free (GF)**-Mice that are raised devoid of all known microbes.

**Gnotobiotic**-Term used to denote GF mice that are now colonized with a defined community of microbes where all members are known such as the Altered Schaedler Flora and the use of synthetic bacterial communities.

**Specific Pathogen Free (SPF)**-Conventional mice that are devoid of particular known pathogens such as bacterial, viral, fungal, and parasitic inhabitants that could affect the health of the mouse colony and the validity of experimental outcomes.

**Conventional mice**-Laboratory mice that are raised in the presence of a gut microbiome, and are not necessarily considered free of pathogens (they may have inhabitants such as murine norovirus and *Helicobacter hepaticus*) but are generally considered healthy.

**Wild mice**-These are mice that have been captured in a non-lab environment (in the wild) and then transferred to a lab for study. Such mice can be mated with other wild-caught mice for study over several generations. Inbred lines of mice harboring wild-mouse microbiomes, often referred to as “WildR mice” (Rosshart et al., 2017), can be generated through microbial transfer from wild mice to inbred strains of lab mice to limit the effects of genetic variation.

**Synthetic communities**-Communities constructed from cultured isolates from naturally occurring complex microbiomes. These communities may represent isolates from a single donor, or isolates obtained from many different donors. Moreover, they may be constructed from subsets of all cultured isolates.

### Model Gut Microbiomes

**FIGURE 1:**
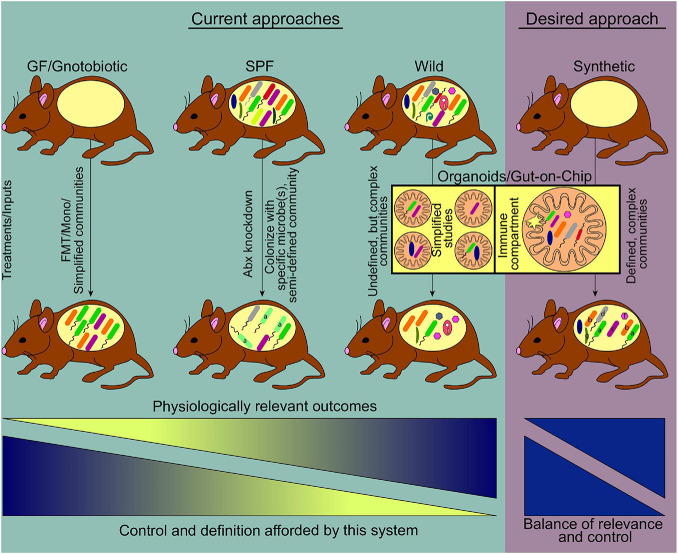
Current and emerging systems to study host-gut microbiome interactions. Prevalent model systems in use to study physiological effects of gut microbiome-host interactions and representative community complexity within each model are shown (teal shaded area). The general usefulness of these systems based on physiologically relevant outcomes, increases left to right. At the same time, the ability to manipulate or define the community structure of these models decreases along the gradient, driving the need for a medium where physiologic relevance and tractability reach an optimum. Current technologies striving toward this desired model include the use of germ free mice where synthetic communities of known microorganisms are used for colonization, as well as the use of emerging lab-on-chip approaches (pink shaded area) (FMT, fecal microbiome transplant; Mono, monocolonization; Abx, antibiotics).

To counteract the effects of microbiome variation researchers have turned to the use of defined reference communities. Beginning in the 1960s, Dubos et al. (1965), Schaedler et al. (1965), and Dewhirst et al. (1999) developed small model communities that were used to standardize the microbiome of animal models, mostly to be used in conventionally raised mice (Box 1). In an effort to move toward systems with greater control over community composition an ever-increasing number of researchers have begun to adopt germ-free/gnotobiotic models first established more than 60 years ago (Trexler and Reynolds, 1957; Gordon and Pesti, 1971). Such models allow the study of communities of interest without unwanted invasion by microbes present in the environment. Pioneering studies using small communities, ranging from mono-associations (colonization with a single microbe) that establish the roles of individual genes and metabolic pathways in bacteria (Rey et al., 2010; Koppel et al., 2018; Glowacki et al., 2020) to more complex communities with up to 20 members (Mahowald et al., 2009; Faith et al., 2011, 2014; Geuking et al., 2011; Cahenzli et al., 2013; McNulty et al., 2013; Sefik et al., 2015; Brugiroux et al., 2016; Geva-Zatorsky et al., 2017; Becattini et al., 2021), reduced the complexity of the system (Figure 1). These simplified models have provided high-resolution insights into the ecological, transcriptional, and metabolic responses of microbes to environmental variations (e.g., diet, inflammation) (McNulty et al., 2013; Ridaura et al., 2013; Becattini et al., 2021), uncovered microbe-microbe interactions that shape community function (Mahowald et al., 2009; Caballero et al., 2017), and highlighted a role for microbiome members in host growth (Blanton et al., 2016; Charbonneau et al., 2016; Schwarzer et al., 2016), weight gain and metabolic health (Fei and Zhao, 2013; Ridaura et al., 2013), pathogen resistance (Fukuda et al., 2011; Hsiao et al., 2014; Alavi et al., 2020), as well as specific enzymatic functions that directly impact host health (Skye et al., 2018; Song et al., 2020). Thus, model communities studied in germ-free/gnotobiotic mice have provided key insights into the several facets of microbiome function and host-microbiome interactions.

The power of the germ-free mouse as a model system may be best exemplified by the advances they have facilitated in understanding immune-microbiome mutualism (reviewed extensively in Honda and Littman, 2016; Ost and Round, 2018; McCoy et al., 2019; Ahern and Maloy, 2020; Ansaldo et al., 2021). These studies have revealed the profound impact of the gut microbiome on immune function and provided detailed insights into the mechanisms that underlie these interactions. The absence of a microbiome leads to the development of a drastically altered intestinal immune system with striking defects in adaptive immune function including reductions in lymphocyte numbers and activation within the intestine and mesenteric lymph nodes, and an enhanced susceptibility to infection by certain pathogens (Abrams and Bishop, 1966; Thompson and Trexler, 1971; Imaoka et al., 1996; Macpherson and Harris, 2004). Notably, the microbiome has also been implicated in the shaping of the innate immune compartment, including aspects of trained immunity (reviewed in McCoy et al., 2019; Negi et al., 2019). Intensive efforts have subsequently identified specific microbial taxa/microbial consortia, and their derived molecules that influence the intestinal immune system of gnotobiotic mice, especially the intestinal T cell compartment. Colonizing germ-free mice with microbial consortia like the Altered Schaedler flora (ASF) and select *Clostridium* species, or mono-colonization with specific members of the *Bacteroides* genus (*Bacteroides thetaiotaomicron*, *Bacteroides caccae*, and *Bacteroides fragilis*) or *Bifidobacteria* (*B. bifidum*), coordinates the development of colonic regulatory T cells, an anti-inflammatory population of CD4^+^ T cells that maintain intestinal homeostasis (Round and Mazmanian, 2010; Atarashi et al., 2011, 2013; Geuking et al., 2011; Faith et al., 2014; Sefik et al., 2015; Verma et al., 2018; Wegorzewska et al., 2019). Furthermore, cellular products derived from these microbes, like polysaccharide A (PSA) (Round and Mazmanian, 2010) or β-glucan/galactan polysaccharides (Verma et al., 2018), short-chain fatty acids (SCFA; gut microbial fermentation products) (Arpaia et al., 2013; Furusawa et al., 2013; Smith P. M. et al., 2013) or microbially-transformed bile acids (Song et al., 2020) also regulate the size and function of colonic regulatory T cell pools. Likewise, Th17 cell differentiation can be coordinated by distinct microbes, most notably segmented filamentous bacteria (SFB) (Gaboriau-Routhiau et al., 2009; Ivanov et al., 2009) in addition to *Bifidobacterium adolescentis* (Tan et al., 2016) or particular strains of *E. coli* (Britton et al., 2020), which in turn can improve protective immunity against pathogens (Ivanov et al., 2009). Intraepithelial T cell populations are also impacted by gut microbes, with SFB able to shape the activation status of this immune compartment (Umesaki et al., 1999), while *Lactobacillus reuteri* promotes the development of CD4^+^ CD8αα ^+^ intraepithelial lymphocytes (IELs) by agonizing the aryl hydrocarbon pathway (Cervantes-Barragan et al., 2017).

Despite the enormous power of these systems, they have several limitations. First, the physiologic relevance of model communities can be hard to decipher. Second, due to their low diversity, they may fail to identify redundancy in effector functions that exist in larger communities, inappropriately attributing essential roles to particular microbes. Third, most groups study a limited number of strains of each species, which ignores the enormous strain-level variation present in the gut microbiome. To overcome these limitations, models where mice are colonized with human gut microbiomes from healthy or diseased individuals (“humanized” mice) that could transmit features of their donor’s health status (Raibaud et al., 1980; Turnbaugh et al., 2009b; Ridaura et al., 2013; Smith M. I. et al., 2013; Kau et al., 2015; Berer et al., 2017; Cekanaviciute et al., 2017; Britton et al., 2019; Feehley et al., 2019) have been widely adopted. The microbiomes of such humanized mice are typically more diverse than model communities and comprising distinct strains depending on the individual donor. Moreover, they represent a system with clear translational relevance, albeit with less defined community membership. For example, microbiome transplantation from individuals suffering from IBD (Britton et al., 2019), food allergy (Feehley et al., 2019), undernutrition (Smith M. I. et al., 2013; Kau et al., 2015; Wagner et al., 2016), obesity (Ridaura et al., 2013), and multiple sclerosis (Berer et al., 2017; Cekanaviciute et al., 2017), among other conditions, could enhance the susceptibility to such diseases in recipient gnotobiotic mice. However, despite all their advantages, significant caveats to the use of human-derived microbiomes exist that suggest a need for new and improved approaches. First, these communities are typically neither defined nor cultured (with exceptions; Wagner et al., 2016; Britton et al., 2019, 2020), limiting the establishment of causality for specific microbes and/or microbial products. Second, while it is clear that microbes derived from humans can modulate particular facets of the murine immune system, host-specificity in such interactions (Atarashi et al., 2015) means that human-derived microbiota may not shape immune responses equivalent to murine-derived microbes (Gaboriau-Routhiau et al., 2009; Chung et al., 2012; Lundberg et al., 2020). Third, human-derived microbiomes are not as well adapted to the murine intestine as mouse-derived communities. Elegant studies using gnotobiotic mice colonized with either human or murine-derived microbiomes demonstrated that the murine microbiome exhibited superior fitness in the mouse intestine, and could displace many members of a human microbiome from a stably colonized mouse upon co-housing (Seedorf et al., 2014). Finally, the overall community composition and structure in the recipient mouse may vary significantly from the donor, both in terms of membership and microbial abundance, potentially over or understating the contributions of particular microbes (Turnbaugh et al., 2009b; Chung et al., 2012; Krych et al., 2013; Xiao et al., 2015; Lundberg et al., 2020). While there is obvious value to these approaches, and a wealth of information has been obtained from their use, it is important to consider that key elements of host-microbiome interactions may be missed by studying microbes outside of their natural environment (i.e., human microbes in the mouse).

While no single system is perfectly suited to address all goals, an optimized model to study host-microbiome interactions should encompass as many of the following features as possible: (i) Completely defined microbiome with high-quality reference genomes for each organism. Such systems allow high-resolution strain quantification and gene expression profiling with strain-level gene expression assessment; (ii) culturable and genetically manipulable strains. If all strains have been captured in culture in a clonally arrayed format, it allows for the construction of consortia of defined membership to determine how specific members impact the phenotype being studied (Goodman et al., 2011; Ahern et al., 2014; Faith et al., 2014), commonly referred to as synthetic communities. Moreover, the availability of tools for genetic modification allows for unambiguous assessment of the role for genes of interest, which has contributed to the significant insights afforded by members of the *Bacteroides*, for which sophisticated tools are available (Anderson and Salyers, 1989; Koropatkin et al., 2008; Goodman et al., 2009; Mimee et al., 2015; Wu et al., 2015; Lim et al., 2017); (iii) a genetically tractable host that allows for the interrogation of host pathways that mediate the effects of specific microbes/microbial products; (iv) a germ-free host that allows for high-level control over the composition of the community. The utility of germ-free mice for advancing microbiome studies is hard to overstate, and coupled with the ability to generate host mutants to dissect pathways of host-microbiome interactions is invaluable; and (v) microbiome whose members and imprinted host responses mirror the human population being modeled. Such a system would allow a high-resolution examination of complex communities that imprint human-like phenotypic variation, overcoming the shortcomings of the systems currently in vogue. An idealized system will capture all the advantages of the systems described above and is represented in Figure 1. While a model that captures all these parameters remains aspirational, advances in microbial culturing and isolation, allied to genomic approaches that continue to decrease in cost while increasing in output mean that large libraries of cultured isolates can now be generated and characterized in wild-type and genetically modified gnotobiotic mice.

One of the primary challenges has been the identification of murine microbiomes that promote human-like phenotypes. Recent ground-breaking studies from a small number of labs have revealed that the microbial inhabitants of mice in the wild (or in pet stores) promote the acquisition of a human-like immune system in lab mice (Figure 1), recapitulating the activated and antigen-experienced phenotype found in humans (Beura et al., 2016; Rosshart et al., 2017, 2019; Lin et al., 2020; Yeung et al., 2020) by contrast with the typical immune system of lab mice that has a phenotype more akin to that of neonates (Beura et al., 2016). Wild mouse microbiomes lead to a profound reshaping of the host immune system, promoting a more human-like phenotype, most potently with respect to boosting T cells with effector/memory phenotypes. These alterations in immune phenotype are characterized by an expansion in systemic and tissue-resident memory CD8^+^ T cell populations, increases in effector CD4^+^ T cells (Th1, Th2, Th17, and Tregs), and innate immune populations such as innate-lymphoid cells and neutrophils (Beura et al., 2016; Lin et al., 2020; Yeung et al., 2020). Moreover, the levels of serum immunoglobulins and select cytokines are also increased (Beura et al., 2016). Consistent with this, these animals were found to be more resistant to viral infections (Influenza A), bacterial infections (*Listeria monocytogenes*), and colon cancer (DSS plus azoxymethane model) (Beura et al., 2016; Rosshart et al., 2017). Conversely, they are more susceptible to surgery-associated sepsis, likely due to increased inflammatory tone and/or increased reactivity to microbial products (Huggins et al., 2019). Thus, wild microbiome-driven enhancement of host immunity is associated with improved immune-mediated resistance to a variety of infectious diseases and cancer, and enhanced susceptibility to other inflammatory diseases, linking microbiome-mediated immunomodulation to organismal health.

Notably, the transcriptional responses that distinguish neonates and adults are reminiscent of those that differentiate SPF mice and those harboring a wild/pet store microbiome. These features are communicable to lab mice following co-housing, suggesting they are microbially-driven (Beura et al., 2016; Rosshart et al., 2017), although whether this is attributable to pathobionts, or the distinct strain composition of these communities is unknown. Moreover, the genomic diversity of wild mice, and its impact on host immune responses and disease susceptibility, may additionally contribute to distinct phenotypic features of wild mice, representing an area ripe for further exploration. Some of these features are likely attributable to infectious agents like pathogenic viruses (Reese et al., 2016), but notably, may also be due to specific endogenous bacterial or fungal members of the gut microbiome (Lin et al., 2020; Yeung et al., 2020). Indeed, wild mice that retained SPF status (i.e., free of all pathogens excluded under SPF guidelines) also induced distinct phenotypic variation relative to lab-raised SPF counterparts, and this could be transmitted to lab mice through gut microbiome transplantation (Rosshart et al., 2017). These data suggest that differences in the specific strain composition of the gut microbiome of wild mice, rather than pathogen exposure, are responsible. More recently, a study revealed that wild mice more faithfully recapitulate the outcome of clinical trials targeting the immune system (Rosshart et al., 2019), by contrast with their conventional lab animals, reinforcing the notion that such models are of greater translational relevance. Thus, wild mice microbiomes provide an opportunity to improve the utility of mouse model systems.

However, while we and others (Hamilton et al., 2020; Graham, 2021; Kuypers et al., 2021) posit that wild microbiome-elicited phenotypes create a murine system with a more human-like phenotype, different approaches to wilding the microbiome including the specific donor material used, or the creation of environments that more accurately mimic the natural environment of the mouse (Arnesen et al., 2020; Lin et al., 2020; Yeung et al., 2020) vs. co-housing under controlled laboratory conditions (Beura et al., 2016; Rosshart et al., 2017), may lead to disparate phenotypic outcomes in recipients. The use of wild microbiomes thus may not fully humanize the murine response or be fully representative of human phenomena. For example, in the case of allergy development and the hygiene hypothesis, one study (Ottman et al., 2019) suggests that lack of microbe diversity drives such allergic states, while (Ma et al., 2021) contradicts these claims. Undoubtedly, the approach of using wild mice and/or their derived microbiomes does not fully address the nuances of *in vivo* mouse models vs. humans as discussed in depth elsewhere (Hamilton et al., 2020; Graham, 2021; Kuypers et al., 2021). To address these limitations, more studies are needed with comparative phenotyping of adult human and wild microbiome-exposed murine immune systems to determine whether these responses truly reflect the development of a more human-like response, as opposed to a response that is simply distinct from lab mice. With the advent of approaches that allow for detailed assessment of the non-lymphoid immune compartment in humans (Szabo et al., 2019), such studies are now possible. Moreover, as we discuss below, detailed knowledge of the microorganisms that coordinate the phenotypic features shared between mice harboring wild microbiomes and humans will advance efforts to generate improved murine model systems.

### Synthetic Wild Communities, an Optimized System to Study Immune-Microbiome Interactions

While wild microbiomes offer the potential to shed new light on host-microbiome interactions, currently there is limited information regarding the effector microbes within these communities. There is therefore a need to determine which microbes are responsible for mediating the human-like phenotypic variation that they induce. These efforts need not focus solely on non-pathogenic members of the microbiome but should include controlled pathogen as well as non-pathogen exposures. While defining these effector microbes is a daunting challenge, we and others have described effective strategies to do so in a systematic and efficient manner (Goodman et al., 2011; Ahern et al., 2014; Faith et al., 2014; Palm et al., 2014; Kau et al., 2015; Surana and Kasper, 2017). The generation of culture libraries in arrayed format (i.e., where individual wells of multi-well plates contain distinct microbiome members) from human donor microbiomes that retain the effector functions of the donor community has allowed mechanistic insight into host-microbiome interactions of biological relevance (Ridaura et al., 2013; Faith et al., 2014; Wagner et al., 2016). Such strategies allow the precise delineation of the effects of individual community members, whether they operate in concert with other members or in isolation (Ahern et al., 2014; Faith et al., 2014). Similarly, the isolation in pure culture of the constituents of the wild mouse microbiome represents a key first step toward the generation of more complete synthetic communities that recapitulate wild microbiome imprinted functions and allow for greater manipulability. Likewise, in defining these wild microbiomes, it is important to appreciate that for the past several decades, non-bacterial residents of the gut (fungi, archaea, viruses, parasites, and other non-fungal eukaryotic members) have received scant attention, mostly attributable to technical challenges. Such impediments include the variability of internal transcribed spacer regions (ITS) within fungal ribosomal genes and a general lack of reference genomes to compare species prevalence in metagenomic samples (Paterson et al., 2017). Sequencing challenges have also dampened the ability to get a fully representative picture of the gut virome. Most techniques for nucleic acid isolation and sequencing are biased toward DNA viruses, largely missing RNA viruses, while isolation and propagation of gut viruses is also a challenge that needs to be overcome (Wang, 2020; Khan Mirzaei et al., 2021). This lack of cultivation methods extends to archaeal members as well, with archaea often requiring specific culture conditions (Borrel et al., 2020) which can hamper *in vivo* studies. The genesis of libraries of isolates of all microbiome-member types in arrayed format from wild-microbiomes that are known to impact host phenotypes, such that consortia of individual library members can be compiled to study their effects on the host will form an essential component moving forward in defining complex host-microbiome interactions (Goodman et al., 2011; Ahern et al., 2014; Faith et al., 2014; Palm et al., 2014). Moreover, this will facilitate the dissemination to other researchers for implementation in their studies. The use of a common library of microorganisms freely available to all researchers that can be leveraged to understand host-microbiome interactions at multiple scales will advance efforts to uncover mechanistic insights into the operations of large diverse communities that reflect the breadth of microbial taxa and viruses that characterize humans and their associated phenotypic effects.

Despite our call for more standardized gut microbiomes, it would be foolish to demand complete homogenization across institutions, or even within an institution. Microbiome variation can itself represent a form of “natural experiment” that can present a challenge to researchers, but that has also proved a rich source of information regarding how the microbiome can mediate interpersonal variation among a population. For example, the varied presence of Th17 cells in the small intestinal lamina propria in C57BL/6 mice from different commercial vendors led to the identification of a single microbe, SFB, which was differentially represented in the microbiomes of these animals and was both required and sufficient for the development of intestinal Th17 cells (Ivanov et al., 2008, 2009; Gaboriau-Routhiau et al., 2009). Similarly, the varying presence of *Lactobacillus reuteri* in different animal facilities within the same institution led to its identification as a potent modulator of CD4^+^ CD8αα^+^ IEL development (Cervantes-Barragan et al., 2017). More recently, the fungus *Debaryomyces hansenii* was highlighted as a mediator of impaired intestinal healing, which again was differentially represented among different colonies of lab mice at the same institution and directly regulated the intestinal healing potential of mice (Jain et al., 2021). Others have also linked different microbiome composition to the phenotype of various animal models of infectious and autoimmune disease (Wu et al., 2010; Lee et al., 2014; Hilbert et al., 2017; Moskowitz et al., 2019; Velazquez et al., 2019). What these studies highlight is that phenotypic variance can be leveraged, even embraced (Ivanov et al., 2008, 2009; Cervantes-Barragan et al., 2017; Jain et al., 2021), to uncover novel host-microbiome interactions that shape host responses. Consequently, although variation poses challenges for microbiome research, total standardization is itself not without issue. Instead, the utility of such variance is linked to an ability to measure and define the causes of the variation, and the reporting of the microbiome composition that is associated with a phenotype will be of enormous value in linking specific microbes to phenotypes of interest. Such an approach will reveal contextualized roles for host phenotypes that may manifest only in the presence of particular community types. Thus, there remains a prominent place for non-standardized models in illuminating fundamentally important host-microbiome interactions.

### Alternatives to *in vivo* Mouse Models

In spite of all the advantages of the *in vivo* mouse models we describe, it is ultimately a system with limitations that demands alternative approaches that augment our understanding. Fundamental differences between mice and humans mean that key aspects of host-microbiome interactions may not be modeled in a murine system. Indeed, the specificity in molecular aspects of host-microbe interactions (Lecuit et al., 1999; Atarashi et al., 2015) demands systems to study human-derived microbes in the context of human cells. The advent of sophisticated *in vitro*/*ex vivo* approaches that use human-derived cells that can themselves be genetically manipulated represent attractive alternatives that can be used in parallel to murine models. In addition to overcoming shortfalls in murine systems, these approaches help with the continued efforts to replace, reduce, and refine animals in research.

#### Organoids

Human intestinal organoids (HIOs) or enteroids (HIEs) remove the need for a live model organism, and instead rely on primary cells derived from human biopsies or stem cells. This technique was originally pioneered from the use of *ex vivo* tissue explants of human intestines (Browning and Trier, 1969). Growth factors are used to drive differentiation of Lgr5^+^ intestinal stem cells into intestinal cell types that mimic the 3D spatial and functional environment of the intestine, allowing for simultaneous differentiation into discrete cell types (Ootani et al., 2009; Sato et al., 2009). HIOs have some advantages over other *in vitro* systems as they maintain the crypt-villus architecture and allow for multiple columnar cell types to be generated (Hill and Spence, 2017). HIOs permit the study of phenomena that have proved challenging in other *in vivo* systems, including tight junctions of non-enterocyte cells of the small intestine (Pearce et al., 2018); IBD models of infection and inflammatory processes (Angus et al., 2019; Sarvestani et al., 2021); and models of infection such as human norovirus (Ettayebi et al., 2016) and rotavirus (Finkbeiner et al., 2012), for which no *in vivo* model organism exists, and *in vitro* culture efforts had not been successful at the time. Additionally, microinjection of bacterial and parasitic pathogens including *C. difficile* (Leslie et al., 2015), *S. enterica Typhimurium* (Forbester et al., 2015; Wilson et al., 2015), *E. coli* (In et al., 2016; Karve et al., 2017; Rajan et al., 2018), and *Cryptosporidium* (Heo et al., 2018) have all been performed. This platform has allowed for controlled studies into the interactions these pathogens have with the intestine, however, there are several key challenges that remain. Although bacteria can be injected into these structures, the process has drawbacks such that the specialized technique of microinjection is required to avoid compromising the organoid structure and the lumen within the organoid contains a growth-limiting concentration of nutrients that generally can only support the growth of bacteria for less than 24 h. This nutrient limitation also significantly hinders the diversity of microorganisms that can be cultured together in poly-microbial communities. Other constraints involve the physical structure of organoids, and the lack of immune cells, calling into question how well the system recapitulates *in vivo* biology with the absence of such features (Blutt et al., 2018). Despite the advantages that organoids provide, the noted limitations suggest that the organoid model is not yet advanced to the point of being able to replicate all aspects found *in vivo.* Instead, these systems are likely more useful as tools to study parameters such as the permeability of the mucosa, drug kinetics, and bacterial interactions in disease states using tissue derived from patients with IBD or related conditions.

#### Gut-on-Chip Technologies

A relatively new approach to studying microbe-microbe and intestinal cell-microbe interactions are “lab-on-chip” technologies. Although systems such as Transwell plates (two-dimensional technology) and Ussing chambers (three-dimensional) have been used for decades (Ussing and Zerahn, 1951; Hidalgo et al., 1989) and have been used to study bacterial-host epithelium interactions and diseases of the intestine (Kurkchubasche et al., 1998; Thomson et al., 2019), both have known limitations. With respect to the microbiome, these technologies are not well-suited for maintenance of both aerobic and anaerobic compartments except in limited circumstances (Ulluwishewa et al., 2015; Jafari et al., 2016). This makes studying gut microbiome-host interactions with obligate anaerobes a challenge. Additional limitations involve the duration in which bacteria can be co-cultured before either they or the epithelial cells die, with most ranging from hours to a few days due to the non-peristaltic nature of these devices (Sadaghian Sadabad et al., 2015). Although other devices, such as the mucosal simulator of the human intestinal microbial ecosystem (M-SHIME), or its derivative, the Host-Microbiota Interaction (HMI)-module, have shown promise in incorporating peristalsis-like flow; they suffer from similar problems of short co-culture incubation times and rely on artificial mucus layers (Van den Abbeele et al., 2012; Marzorati et al., 2014). The SHIME reactors are also large, expensive to produce, and not easily scalable.

Using technology pioneered by lithography of computer chip manufacturing (Bhatia and Ingber, 2014), microfluidic devices may be a happy medium that yields more information on bacterial-gut epithelial-immune system interactions. These devices allow for a 3-D spatial reconstruction of the *in vivo* environment. Specifically for bacteria, these platforms have been used to study bacterial quorum sensing (Osmekhina et al., 2018), the response to antibiotics, and other chemicals in a complex community (Hsu et al., 2019), and taxis and motility (Gurung et al., 2020). Adoption of these devices has led to the generation of new platforms that are being used to study the human gut ecosystem and the human gut microbiota (von Martels et al., 2017; Tan and Toh, 2020). These devices overcome a substantial amount of the need for using *in vivo* models and tissue explants to maintain an environment necessary to study long-duration, complex, multi-species, and multiple cell type interactions. Although there are at least 12 different microfluidic devices in use, the vast majority rely on the colorectal carcinoma cell line, Caco-2 cells, to establish an epithelium, and have only managed to culture one species of bacteria at a time; several of these devices have previously been reviewed for benefits and drawbacks (Bein et al., 2018; Tan and Toh, 2020). Recent advancements include the *nBio*Chip which supports the co-culture of both bacteria (Staphylococcus *aureus* and *Pseudomonas aeruginosa*) together with the fungus, *Candida albicans* (Srinivasan et al., 2017). Perhaps the largest advancement is the Intestine Chip, with the ability to maintain over 200 operational taxonomic units (OTUs) of bacteria directly from human feces with both obligate anaerobes and aerobic bacteria established along a hypoxia gradient (Jalili-Firoozinezhad et al., 2019). The Intestine Chip can also support stable colonization periods of up to or beyond 1 week due to its peristalsis-like flow of media. However, like the platform of the two previous iterations of this specific device (Kim et al., 2012, 2016), Caco-2 cells are used to develop the epithelial compartment rather than primary cells, which limits some downstream applications due to these cells not being representative of the primary cells of the intestinal tract.

Recent advancements have merged organoid and gut-on-chip technologies. First described as an early version of the Intestine Chip, the use of matured organoids containing villus structures and multiple cell types were enzymatically broken down and used to seed extracellular matrix (ECM)-coated membranes of a microfluidic chip (Kasendra et al., 2018). The other half of the chip was then seeded with human intestinal microvascular endothelial cells to examine cell-cell interactions, thus creating a multi-system organ on a chip. Through RNA-sequencing, confocal microscopy, and tissue staining it was shown that the Intestine Chip recapitulates key features of the signaling pathways, cellular differentiation, mucus production, and epithelial-endothelial interactions seen in the human duodenum. A similar platform termed the gut microbiome physiome (GuMI) has been developed, specifically for the culture of extremely oxygen-sensitive microbes such as *Faecalibacterium prausnitzii* (Zhang et al., 2021). Similar to the Intestine Chip, the gut microbiome (GuMI) ECM is impregnated with cells derived from organoids and the device has inlets for sampling and injection of bacteria. While many other chip-based devices are fabricated using polydimethylsiloxane (PMDS), the GuMI uses polysulfone, which can be autoclaved for sterility and is less permeable to oxygen, allowing for more strict control of oxygen gradients (Shin et al., 2019). Lastly, this chip allows for the independent culture of six different bacteria within the luminal portion. Using organoid-derived cells, this three-layer-chip contains an ECM seeded with both intestinal cells and monocyte-derived macrophages and recapitulates features observed in IBD patients (Beaurivage et al., 2020). While the presence of immune cells is an important advancement, the generation of systems that can maintain interaction with a complex immune system is essential to boost the translational relevance of these systems. Nevertheless, the combination of gut-on-chip and organoids is a promising step forward toward the goal of having a tunable system to interrogate complex interactions that are difficult to perform *in vivo* (Figure 1).

## Discussion/Prospectus

A wealth of knowledge about gut microbiome-host interactions has been gained through the use of the model systems discussed in this review. Conventionally raised mice (both SPF and non-SPF), and gnotobiotic mice, have been and continue to be essential tools to study interactions between the gut microbiome in host health and disease. While there is a continued use for these models, their limitations hinder efforts to gain the mechanistic insights required to target the microbiome for therapeutic purposes. Development of synthetic, wild mouse gut microbiome communities comprising cultured and genome-sequenced microbiome members derived from wild mice provides an opportunity to gain mechanistic understanding of specific microbe-host phenotypes that recapitulate the interactions of humans with their microbiomes and the associated microbiome imprinted phenotypes. Used in conjunction with ever-improving *in vitro*/*ex vivo* model systems that facilitate high-resolution studies of complex host-microbiome interactions, these technologies will advance our understanding of the range of microbiome members that shape host physiology and help define the nature of the interactions that underlie these phenomena.

## Conclusion

In conclusion, model systems to study gut microbiome-host interactions continue to evolve. The incorporation of synthetic, wild microbiomes into the suite of model systems provides an opportunity to increase mechanistic insight and translatability. The use of these advanced mouse models and ever-improving alternative model systems to study gut microbiome-host interactions will increase our understanding of the functionality of specific microbes in human physiology and disease, advancing efforts to target the microbiome for therapeutic purposes.

## Author Contributions

RWPG, MJE, and PPA performed relevant literature searches, critical appraisals of the literature, and wrote the review. All authors contributed to the article and approved the submitted version.

## Conflict of Interest

The authors declare that the research was conducted in the absence of any commercial or financial relationships that could be construed as a potential conflict of interest.

## Publisher’s Note

All claims expressed in this article are solely those of the authors and do not necessarily represent those of their affiliated organizations, or those of the publisher, the editors and the reviewers. Any product that may be evaluated in this article, or claim that may be made by its manufacturer, is not guaranteed or endorsed by the publisher.
